# A Survey of Security Services, Attacks, and Applications for Vehicular Ad Hoc Networks (VANETs)

**DOI:** 10.3390/s19163589

**Published:** 2019-08-17

**Authors:** Muhammad Sameer Sheikh, Jun Liang, Wensong Wang

**Affiliations:** 1 School of Automotive and Traffic Engineering, Jiangsu University, Zhenjiang 212013, China; 2 Department of Automotive and Transportation Engineering, Automotive Engineering Research Institute, Jiangsu University, Zhenjiang 212013, China; 3 School of Electrical and Electronic Engineering, Nanyang Technological University, Singapore 639798, Singapore

**Keywords:** vehicular ad hoc network (VANET), intelligent transportation system (ITS), security threats and attacks, privacy-preserving authentication, cryptography

## Abstract

Vehicular ad hoc networks (VANETs) are an emerging type of mobile ad hoc networks (MANETs) with robust applications in intelligent traffic management systems. VANET has drawn significant attention from the wireless communication research community and has become one of the most prominent research fields in intelligent transportation system (ITS) because of the potential to provide road safety and precautionary measures for the drivers and passengers. In this survey, we discussed the basic overview of the VANET from the architecture, communication methods, standards, characteristics, and VANET security services. Second, we presented the threats and attacks and the recent state-of-the-art methods of the VANET security services. Then, we comprehensively reviewed the authentication schemes that can protect vehicular networks from malicious nodes and fake messages. Third, we discussed the latest simulation tools and the performance of the authentication schemes in terms of simulation tools, which was followed by the VANET applications. Lastly, we identified the open research challenges and gave future research directions. In sum, this survey fills the gap of existing surveys and summarizes the latest research development. All the security attacks in VANETs and their related countermeasures are discussed with respect to ensuring secure communication. The authentication schemes and comprehensive applications were introduced and analyzed in detail. In addition, open research challenges and future research directions were issued.

## 1. Introduction

In recent years, the new development and enhancement in intelligent transportation systems (ITSs) have gained significant attention from both industry and research communities. The ITS contributes the major part in terms of providing road safety, improving traffic flow, and offering entertainment services on vehicles [1]. The automotive industry realizes the need for the vehicles to be connected with the wireless communication system, which enables communication between vehicles and between vehicles and infrastructure. Such communication can significantly increase traffic safety and optimize the traffic flow [2]. It can also broaden the vehicle recognition events, which, at present, cannot be detected by available electronic sensors and loop detectors. Therefore, embedded sensors have been introduced in which traffic information such as driving behaviors, traffic flow parameters, and driving conditions can be shared with nearby vehicles by developing networks, which are named Vehicular ad hoc networks (VANETs) [3]. 

VANET is a type of mobile ad hoc network (MANET) with road routes, which aim to provide traffic safety, improve traffic flow, and enhance the driving experiences. It relies on transportation authorities for registering and managing the roadside units (RSUs) and onboard units (OBUs) [4]. An OBU is installed in every vehicle as a transmitter to communicate with other vehicles on the road, while RSUs are installed along the street with network devices. RSUs are used to communicate with the infrastructure and contain network devices for dedicated short-range communication (DSRC) [5]. VANETs are classified into two categories: vehicle-to-vehicle (V2V) and vehicle-to-infrastructure (V2I) communications [6]. In the V2V, vehicles can communicate with each other in order to exchange traffic-related information. In the V2I, vehicles can directly communicate with the infrastructure to exchange traffic information.

In recent years, significant development and advances in vehicular technology have provide many resources such as storage devices, radio network, robust computational power, and different kinds of vehicle sensors. Wireless sensor networks (WSNs) are the robust technology for the Internet of Things (IoT) and significantly applied in ITS. In this technology, sensors are fixed on the roadside to identify the traffic situations and deliver the traffic-related information to vehicles such as collision warning, lane-changing warning, and more. This traffic information can significantly enhance the traffic and road safety and improve driving experiences. In the ITS context, WSNs can be regarded as an additional element in which they can cooperate with the vehicular network, i.e., VANETs. This integration between vehicular ad hoc and wireless sensor networks forms a hybrid network, termed VANET-WSN [7]. As the number of real-time applications is increased, the quality of service (QoS) for the VANET-WSN has become a prominent research area in the wireless communication technology. Estimation of bandwidth has greater importance for network QoS assurance, which can optimize the channel capacity, channel allocation, network routing, bandwidth allocation, and more [7]. 

IoT is one of the most prominent research fields in wireless communication technology. It is embedded with electronic devices such as sensors to communicate with other hardware devices via the Internet. In IoT technology, security remains a challenging issue, which seeks to protect the hardware devices and network in the IoT system [8]. The IoT technology is being applied in the self-driving vehicles (SDV) for automated and connected vehicles and microgrids energy resources systems. In the microgrid system, all distributed energy resources combine together to provide a better energy solution. The IoT microgrid system depends on the supervisory control and data acquisition (SCADA), and the integration of domains can increase the chances of possible cyber-attacks, which may attack the SCADA control management system and limit the functionality of hardware devices [8]. The low-cost adaptive monitoring technique has been introduced for IoT devices, which can monitor the intensity and data through the network. In this technique, the energy consumption and data volume are significantly minimized [9,10], which can enable IoT devices for maintaining a battery for a long time and alleviate data consumption services [10]. 

The mobile cloud computing (MCC) system is the robust technology in which the drivers can use a mobile phone device to connect the cloud through the Internet. The MCC combines the technology and resources to monitor and identify the traffic safety by processing the sensor data using different mobile architecture systems. However, the computing devices in the MCC have some limitations such as battery power consumption, resources, and more [11]. 

Challenges and benefits in the ITS have motivated researchers to introduce and promote the vehicular cloud computing (VCC) [12]. The VCC is a new technology, which takes advantage of the cloud computing to offer better services and improve the driving experience of VANET drivers. It aims to provide the services to the drivers, improve traffic flow, reduce traffic congestion, accident, and ensure the usage of real-time software and infrastructure with the QoS [13]. Specifically, the VCC can be the platform for the convergence of ITS and computing and storage capabilities of the MCC. Moreover, the VCC can incorporate the features of ITS, WSN, and MCC for providing better road safety, improving driving conditions, and securing traffic management systems [14]. The self-driving vehicles and advanced assistance application used for highly automated driving will depend on the V2X communication technology, which are empowered by fog and edge computations [15]. Such computations could enhance the VANET mechanism and take the Internet of Vehicles (IoV) to the next level. In the fog computing, the key management technique has drawn great attention due to its characteristic and reliability for providing a secure channel. This technique can be used in VANETs to form a fog system in terms of roadside units (RSUs) such as edge routers, intelligent traffic lights, and more [16].

VANETs have drawn significant attention from the research community due to offering excellent applications in the intelligent traffic management systems. Robust schemes for implementing the ITS, VANETs, and MANETs are quite different in terms of high node mobility, network architecture, and an unreliable channel. The high mobility and volatility in VANETs are vulnerable to the internal and external network attacks [17]. These attacks raise several challenges in order to design secure VANETs: security, privacy, and trust [17]. 

VANETs face many security challenges and issues in terms of authentication and privacy issues [18,19,20,21,22], and untrustworthy vehicles raise many security and communication issues [23]. In VANETs, the entire communication is an open access environment that makes VANETs more vulnerable to the attacks. Thus, the attacker can modify, intercept, inject, and delete the messages. For example, the attacker can get access to the traffic messages, which are used to guide the vehicles on the road. If the attacker alters these messages, it is possible to spread false information on the road and cause traffic congestions, traffic incidents, accidents, and hazards. 

To effectively apply the VANETs in the wireless communication technology, the security and privacy issues must be handled efficiently by designing a sophisticated algorithm, which can tackle all kinds of threats and attacks. To address these issues, several research studies have proposed the related authentication and privacy schemes. Some methods of them utilize the public key infrastructure (PKI) scheme to authenticate vehicles, which contains the digital signature of the certification authority (CA) and vehicles public keys. While the vehicles and RSUs require a large amount of computational time and memory to process and verify these certificates [2,24,25]. These schemes create more robust solutions by verifying signatures of each vehicle. It causes two problems [21]. First, it cannot verify all signatures in limited time due to less power in OBU. Second, each message contains signatures and certificates, which may increase the packet size and then subsequently increase the transmission overhead.

The rest of this survey paper is structured as follows. Section 2 presents the relevant surveys referring to VANETs’ security and privacy challenges as well as the authentication techniques. We explain the overview of VANETs in Section 3. Section 4 presents the security services of VANETs and their threats and attacks. Section 5 presents the recent state-of-the-art methods of VANETs’ security services and privacy-preserving authentication schemes. Simulation tools and applications of the VANETs are discussed in Section 6 and Section 7, respectively. Section 8 presents the open research challenges in VANETs. Lastly, Section 9 concludes the review.

## 2. Relevant Surveys

Many surveys on the security services and privacy schemes for VANETs have been reported. They mainly focus on the different aspects of vehicular communication, standardization, and security services. However, this survey comprehensively covers the VANETs security services, threats, attacks, recent state-of-the-art methods, privacy-preserving authentication schemes, applications, and open research challenges. Hartenstein and Laberteaux [26] presented a tutorial survey in terms of communication and networking aspects of VANETs. This survey covers the potential applications and their requirements by using the communication platform. Then, they discussed the design challenges for VANETs and identified security and privacy challenges in VANETs. Lastly, they discussed existing research challenges in VANETs. In Reference [27], Lin et al. presented a survey on VANETs’ security. In this survey, the authors reviewed the standardization process of VANETs in terms of security services. Then, they identified two issues, i.e., certificate revocation and privacy-preserving, which required a sophisticated mechanism in order to make the standards practical.

In Reference [28], Isaac et al. presented a survey in which they comprehensively discussed the threats and attacks in VANETs and their related countermeasure. Riley et al. [29] presented a survey on existing authentication solutions for VANETs in which they classified the authentication schemes based on some specific criteria, and compared the advantages and disadvantages of these schemes in different conditions. Karagiannis et al. [30] presented a survey on vehicular networks by discussing the basic characteristics, applications, challenges, and their proposed solutions. In Reference [31], Al-kahtani launched a survey on different security attacks in VANETs. This survey discussed the existing security attacks and their related countermeasure. Then, they analyzed and discussed possible future attacks and some future research directions. Zeadally et al. [18] presented a survey on wireless access standards of VANETs and discussed the deployment of VANET technology in the U.S., Japan, and European countries. Then, the authors identified some VANETs’ simulation tools and discussed their advantages and disadvantages. Lastly, they discussed some open research challenges that the researcher faced for deploying VANETs’ technologies. 

In Reference [1], Nidhal et al. launched a survey on the VANET communication architecture, outlined the security and privacy challenges, and classified them in terms of cryptography schemes. Additionally, Al-Sultan et al. [32] presented a survey on the VANET architecture, and outlined simulation tools to simulate VANET protocols and its applications. Vijayalakshmi et al. [33] reviewed some security and authentication techniques for VANETs along with the various kinds of routing protocols to provide secure and efficient communication in VANETs, respectively. In Reference [34], Sharef et al. presented the routing characteristics and challenges in VANETs, which is applicable for designing various kinds of routing protocols. Richard et al. [17] presented a survey on the security issues and challenges of VANETs along with the security requirements, threats, and different kinds of architecture, which were proposed to resolve the security issues. Qu et al. [19] presented the general secure process and outlined the authentication methods involved in these processes. Additionally, the authors performed a detailed survey on authentication algorithms, then reviewed the privacy-preserving techniques, and discussed the trade-off between security and privacy.

Azees et al. [35] presented the basic architecture, characteristics, and security challenges of VANETs. Then, they discussed the attacks on security services along with the possible solutions to ensure secure communication in VANETs. Hasrouny et al. [4] presented the recent development of VANET security challenges and related solutions. This survey outlines the characteristics, challenges, and security requirements in VANETs. Then, the authors discussed the security attacks and their related solutions, and then compared them based on well-known security criteria in VANETs. Kerrache et al. [36] presented a comprehensive survey on trust management for vehicular networks. They discussed the existing trust models along with the comparison between cryptography and trust schemes. Then, they indicated some critical conditions in which existing trust models cannot perform well, and also discussed their possible solutions. 

Manvi and Tangade [37] presented the authentication schemes to provide secured communication in VANETs. This survey discussed the authentication schemes in three categories, i.e., cryptography technique, message verification, and digital signatures techniques. Lastly, the authors identified the open research challenges in the VANET authentication. Sakiz and Sen [38] presented a survey on the attacks and its possible detection mechanism. The attacks are described and classify with their effectiveness, and the solutions to deal with these attacks are discussed with their merits and demerits. Rahim et al. [39] presented a survey on the social aware applications of vehicular social networks (VSNs), data dissemination, and mobility modeling. Then, they discussed the system recommendation, cloud computing, and future research directions. Boualouche et al. [40] launched a comprehensive survey on pseudonym changing strategies for VANETs. Then, they discussed and compared these strategies based on some relevant criteria. Lastly, the authors identified some open research challenges and future research directions. Sharma and Ajay [41] presented an intrusion detection systems (IDSs) and security mechanisms in the vehicular network, i.e., VANETs and VANET cloud. This survey outlines the brief introduction of IDSs and discussed their related schemes in detail. Then, they outline the open research challenges in IDS and identify future research directions. 

In Reference [42], Wang et al. presented a survey on vehicle-to-everything (V2X) application requirements and outlines the need of testing challenges along with the brief discussion on testing methods of V2X communication in terms of the architectural point of view. In Reference [43], Sewalker and Seitz have discussed the design parameters of the vehicle-to-pedestrian (V2P) system, and outlined the existing V2P schemes for safety applications and their design considerations, along with the integration challenges of vulnerable road users (VRUs) with the V2X system. Zhaojun et al. [44] discussed the recent developments in vehicular networks and outlined the security and privacy issues. This survey discussed the basic architecture and characteristics in VANETs. Then, they discussed the security services along with threats and attacks. Second, the authors described the security and privacy issues along with the trust models in VANETs. Ikram et al. [45] launched a survey on authentication and privacy schemes for VANETs in which the authors discussed and classified the security mechanism of these schemes. Then, they indicated some open research issues and security challenges in VANETs.

The above surveys comprehensively cover the VANET architecture and security challenges. However, this survey focuses on the VANET security services and authentication schemes in detail. First, we discuss the VANET security services. Second, we present each security service of VANETs in terms of threats and attacks along with the recent state-of-the-art methods that indicate the existing issues of VANET security and its solutions, whereas the authors in References [36,37,44,45] did not cover these. Furthermore, we comprehensively cover the authentication schemes in detail, while most of the surveys did not cover the authentication schemes except Reference [44], which explained the authentication schemes in detail. Third, the latest simulation tools and the performance of the authentication schemes in terms of simulation tools were discussed, which was followed by the VANET applications, while most of the surveys did not discuss the simulation tools and its utilization on the authentication schemes along with the VANET applications. Lastly, we identified some open research challenges in VANETs that the researcher may face while conducting the research in order to deploy VANET technologies and services efficiently, while most of the previously mentioned surveys did not discuss the existing research challenges in VANETs. In addition to open research issues, we also identified some possible solutions to deal with these issues along with the future research directions. 

## 3. Basic Overview of VANETs

**Figure 1:**
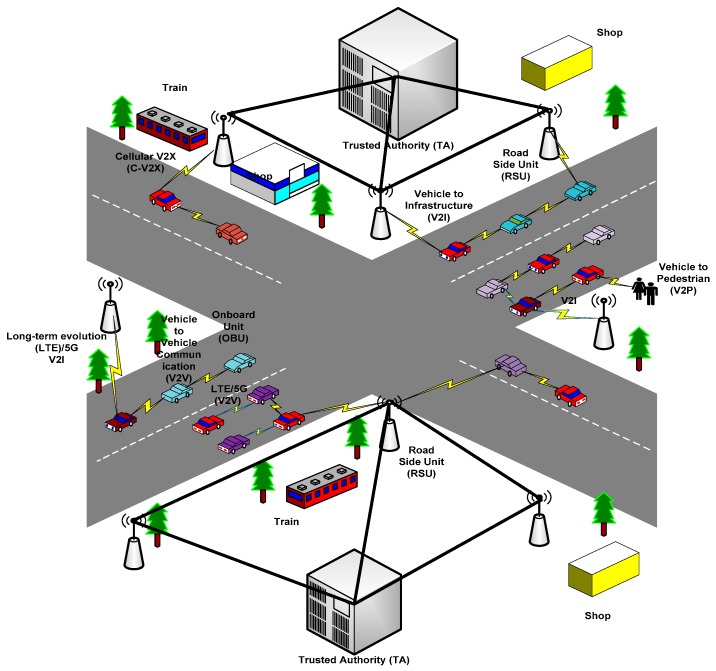
VANET model diagram.

Since 1980, the VANETs, which are ad hoc network infrastructures, grew abruptly, in which vehicles were connected through wireless communication [46]. Recently, VANETs are used to enhance traffic safety, improve traffic flow, reduce traffic congestion, and enhance driving experiences [47]. Figure 1 shows the basic model diagram of VANETs, which consists of three components such as roadside unit (RSU), onboard unit (OBU), and trusted authority (TA). First, we discuss the VANET architecture, i.e., RSU, OBU, and TA. Second, we discuss the communication methods in VANETs, which is followed by the VANET standards. Lastly, we explain the VANET characteristics. 

### 3.1. VANET Architecture

Generally, the communication between vehicles and RSU is done via the wireless technology called wireless access in the vehicular environment (WAVE). WAVE communication ensures the safety of passengers by updating vehicle information and traffic flow. It enhances the pedestrian and driver safety. It also improves the traffic flow and efficiency of the traffic management system. The VANET comprises of several units such as OBU, RSU, and TA. Specifically, the RSU typically host an application that is used to communicate with other network devices, and OBU was mounted in each vehicle to collect the vehicle useful information such as speed, acceleration, fuel, and more. Then, the information is forwarded to the nearby vehicles through a wireless network. All roadside unit (RSU) interconnected with each other is also connected to TA via a wired network. Additionally, TA is the head among all components, which is responsible for maintaining the VANETs [35].

- Roadside Unit: The RSUs are computing devices, which is fixed alongside of the road or in specified location such as: parking area or at the intersection [32]. It is used to provide local connectivity to the passing vehicles. The RSUs consists of network devices for dedicated short range communication (DSRC) based on IEEE 802.11p radio technology. Specifically, RSUs can also be used to communicate with other network devices within the other infrastructure networks [32].
- Onboard Unit: An OBU is a GPS-based tracking device that is usually equipped in every vehicle to sharing vehicle information to RSUs and other OBUs. OBU consists of many electronics components such as: resource command processor (RCP), sensor devices, the user interface, and read/write storage for retrieving storage information. The main function of OBU is to connects with RSU or other OBUs through wireless link of IEEE 802.11p [48], and is responsible to communicate with other OBUs or RSUs in the form of messages. Moreover, OBU take input power from the car battery, and each vehicle consists of a sensor type global positioning system (GPS), an event data recorder (EDR), and forward and backward sensors, which are used to provide input to OBU [35].
- Trusted Authority: TA is responsible for managing the entire VANET system such as registering the RSUs, OBUs, and the vehicle users. Moreover, it has the responsibility to ensure the security management of VANETs by verifying the vehicle authentication, user ID and OBU ID in order to avoid harm to any vehicle. The TA utilizes a high amount of power with large memory size and can also reveal OBU ID and details in case of any malicious message or suspicious behavior [49]. In additional to these, TA has the mechanism to identify the attackers as well.

### 3.2. Communication Methods

The ITS is consistently focused on providing secure communication to improve the traffic flow, road safety, and also overcome the traffic congestion by utilizing different networking techniques such as MANETs and VANETs.

V2X communications play an important role in the ITS to improve the traffic efficiency, traffic safety, and enhance driving experiences by providing real-time and highly reliable information such as collision warning, road bottlenecks information, traffic congestions warning, emergency situations, and other transportation services [50]. V2X communication can exchange the information between V2V, V2I, and vehicle to pedestrians (V2P), as shown in Figure 1. 

In V2V communication, the transmission medium is characterized by a high transmission rate and short latency [4,51]. In the V2V, a vehicle can broadcast useful information such as emergency braking, collision detection, and traffic conditions among each other. The V2I is used to transmit useful information between vehicles and network infrastructures. In this domain, the vehicle developed a connection with RSUs to exchange information with other networks such as the Internet. Furthermore, due to communication with the infrastructure, the V2I require large bandwidth than V2V but is less vulnerable to attacks [52].

Recently, C-V2X technology was introduced. It is a unified connectivity platform that aims to support V2X communications [53]. The C-V2X is developed within the third generation partnership project (3GPP) and regarded as the robust communication technology that can accomplish V2X communications [54]. It connects each vehicle and enables the cooperative intelligent transport systems (C-ITS) that reduce the traffic congestion and enhance traffic efficiency [55]. 

In the year 2016, 3GPP released its first version to support V2X communications, and the standards are referred as LTE-V, long-term evolution (LTE), and C-V2X [56]. LTE possess robust benefits in V2I communication because of its high data rate, large coverage, and penetration rate [57]. However, in V2V communication, LTE faces many challenging issues due to lack of its centralized structure and limited services to support V2V communication [57]. The VANET communication is further classified into four categories [58], which are shown below.

- Warning Propagation Message: If there is any crucial situation where the message is required to send to a specific vehicle or to a group of vehicles. For example, if there is any accident or collision, then a warning message should be sent to the vehicles that are on the way to avoid traffic jams and increase traffic safety. To deal with this issue, a new routing algorithm is required, which can be used to send the warning messages to the destination [58].
- V2V Group Communication: In the V2V communication domain, only vehicles that are sharing some of the same features can take part in this communication [58]. This includes vehicles with the same brand or vehicles sharing the same location in the time interval.
- Vehicle Beaconing: Periodically sent, the beacon messages all vehicles, which are nearby and RSUs. These messages contain the speed, velocity, and acceleration of the sending vehicle.
- Infrastructure to Vehicle Warning: To improve the traffic flow and road safety, warning messages are broadcast from the infrastructure via RSUs to all vehicles within its vicinity when a possible accident or collision is detected, especially in the curve route, intersections, or with a narrow road.

### 3.3. VANET Standards

VANET standardization affects all layers of the open system interconnection (OSI) model, which is used as a communication tool and includes all necessary features of all the layers [1]. The dedicated short range (DSRC) communication, WAVE, and IEEE 802.11p are used to designate the full standard of communication protocol to deal with VANETs.

**Figure 2:**
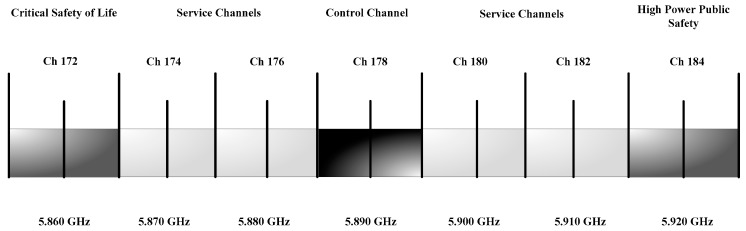
Channel diagram of dedicated short range communication (DSRC).

**Figure 3:**
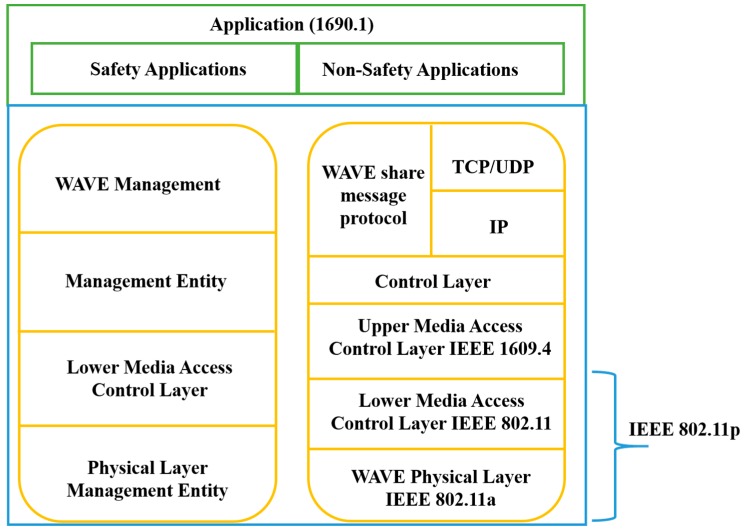
Wireless access in vehicular environments (WAVE) architecture.

- Dedicated Short Range Communication (DSRC): DSRC is a wireless communication technology tool that permits vehicles to communicate with each other in ITS or other infrastructure such as V2V and V2I. In 1999, the Federal communication commission (FCC) allocated the band from 5.850 to 5.925 GHz, with a spectrum of 75 MHz for DSRC [59,60]. As shown in Figure 2, the spectrum of 75 MHz DSRC is sectioned into seven channels, and starts from Ch 172 to Ch 184. The Ch 178 is the control channel that can support the safety power applications [60]. The other six channels such as 172, 174, 176, 180, 182, and 184 are the service channel (SCH). The Ch 172 and Ch 184 are used for high power and public safety messages [60], while other channels can be used to send both as a safety and non-safety messages.
- WAVE: According to the IEEE published materials for the latest ITS standards [61], the WAVE IEEE 1609 describes an architecture, mechanism, sets of protocols, and interface, which are used to develop communication in the VANET environment, i.e., V2V and V2I communications [1]. Figure 3 shows the different standards of the WAVE architecture and its integration with the open system interconnection (OSI) model.
- IEEE 802.11p: After introducing the IEEE 1609 standards, the IEEE extended the family of IEEE 802.11 protocols by adding a new member 802.11p, which is used to facilitate the vehicular communication network [1]. This is in compliance with the dedicated short range communication (DSRC) band.

### 3.4. VANET Characteristics

VANETs are the wireless networks in which nodes are fixed on the roadside units or the high mobility vehicles. It is highly dynamic, reliable, and offers multiple services, but with limited access to the network infrastructure. VANETs have a unique characteristic as compared to MANETs, and these characteristics are very important for security and privacy aspects in VANETs. These VANET characteristics are discussed below.

- High Mobility: High mobility nodes are the main features of VANETs, which have high mobility as compared to MANETs. VANETs are different from other types of ad hoc networks in which nodes can move in random directions, which makes it difficult to estimate their location and network topology, because the vehicles are limited to the road direction, traffic lights, and to respond to other nearby vehicles. In the past, several research studies have explored this special feature [62,63,64]. According to the references [18,65], the high mobility of nodes reduces the communication time between the nodes in the network.
- Wireless Communication: The nodes connection and data communication are exchanged through a wireless medium. Therefore, it is necessary to develop a secure communication during transmission [4].
- Dynamic Network Topology: The topology of VANETs is not constant and can change rapidly due to the high mobility of vehicles. Therefore, it makes VANETs more vulnerable to attacks and difficult to recognize the suspect vehicles.
- Driver Safety: VANETs can improve driver safety, enhances passenger comforts, and improves the traffic flow. The main advantage of VANETs is that vehicles can communicate directly. Therefore, it allows the number of applications, which required directly to communicate between nodes to other networks such as RSUs or OBUs.
- Limitation of Transmission Power: The transmission power is very constrained in the WAVE, which ranges from 0 to 28.8 dBm and was limited to the distance up to 1 Km. Thus, the limited power transmission resulted in limiting the coverage distance of VANETs [66,67].
- Network Strength: The strength of the network in VANETs depends on the flow of the traffic on the street, in case of traffic jams it can be very high, and low when there is no traffic on the road.
- Large Network: The network can be larger in downtown areas, highways, and also the entry and exit point of the city [64,68].
- Volatility: In VANETs, the connections between two nodes are usually developed due to their mobility. Each vehicle has high mobility, so it is possible that the connection among the vehicles can be lost and may remain active within a few wireless hops [44] that makes difficult to ensure the personal security in VANETs.

## 4. VANETs Security and Attacks 

### 4.1. VANET Security Services

Recently, MANETs introduce a new security concern, which was considered an important issue for the researcher to deal with the safety purpose such as fewer central points, mobility, insufficient wireless connectivity, and driver issue [69]. In VANETs, the security ensures that the transferred messages are not injected or altered by attackers. Additionally, the driver is responsible to inform the traffic conditions accurately within the limited time frame. The VANET is more sensitive to the attacks because of its distinctive characteristics. Specifically, security challenges should be addressed properly. Otherwise, it will lead toward creating many constraints for securing communication in VANETs [4]. 

**Figure 4:**
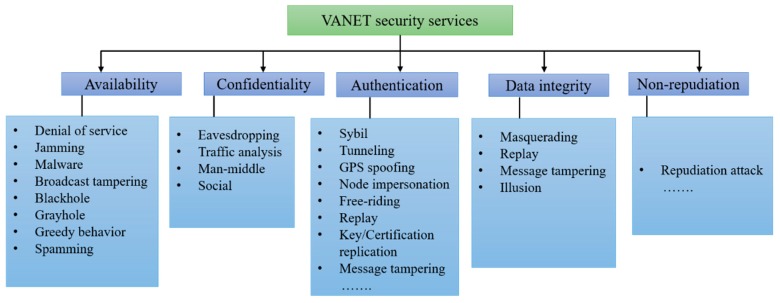
VANET security services.

In the VANET security, it is necessary to mention the requirements that the system should be in-line with the appropriate network operation. Unable to fulfill these requirements may lead to possible threats or attacks in VANETs. The main security requirements are categorized into five main domains such as availability, confidentiality, authentication, data integrity, and non-repudiation [2,70]. Figure 4 shows the security services as well as their threats and attacks. These security services are discussed in this section.

- Availability: Availability is the most important part of security services due to its direct relationship with all safety applications. The main responsibility of availability is managing functionality, and its security must ensure that the network and other applications must remain functional in case of faulty or malicious conditions [71].
- Confidentiality: Based on certificates and shared public keys, confidentiality ensures that the designated receiver have access to the data while outside nodes may not be able to get access to that data until confidential data was received by the designated user.
- Authentication: Authentication plays a vital role in VANET. It prevents the VANET against suspected entities in the network. It is important to have the related information of the transmission mode such as user identification and sender address. Authentication have the right to control the authorization level of vehicles, and it can also prevent Sybil attacks by assigning individual identity to each vehicle [17].
- Data Integrity: It ensures that the message content is not altered during the communication process. In VANETs, data integrity can be ensured by using the public key infrastructure and cryptography revocation process [36].
- Non-Repudiation: It ensures that, in case of the dispute sender, the receiver of the message does not refuse to engage in transmission and reception [72,73].

### 4.2. Security Threats and Attacks in VANETs

VANETs are more sensitive to attacks because of inherent characteristics such as high mobility with frequent disconnection. The network topology of VANETs changes very rapidly due to the high mobility of vehicles. While the nodes are moving at a maximum speed of 20 m/s in MANET simulations, the speeds of the vehicles in VANETs are much faster than this speed limit. Therefore, a communication link breakage between vehicles frequently occurs in the vehicular network. More specifically, the links between vehicles traveling in opposite directions are only limited for a few seconds that lead to frequent disconnection of the vehicular network, and the vehicles can only communicate with each other for a very limited time. Then, the vehicles did not see each other again. Therefore, it makes VANETs more vulnerable to attacks and difficult to recognize the suspect vehicles. 

As the safety messages broadcast in an open access environment, the entire communication in VANETs disturbs when the attacker injects, modifies, or intercepts the messages in VANETs. It makes VANETs more vulnerable to attack because the attacker may fill bogus information in the transmitted message. These challenges expose VANETs to various kinds of dangerous attacks.

In VANETs, the attacker has an intention to disturb the entire network for his own interest. In VANETs, the attackers are classified into four categories [2]. (i) Insider vs. outsider attackers: the insider attackers is the authenticated user with deep knowledge of the network configuration, while the outside attackers are not authenticated users, with limited capability to attack the network than insider attackers, (ii) active vs. passive attackers: active attackers either generate fake messages or do not transmit received messages, while passive attackers did not engage in the communication, (iii) malicious vs. rational attackers: the main aim of malicious attackers to destroy other nodes on the network without obtaining any personal benefits, while the attackers attacked the network to obtain personal benefits, and (iv) local vs. extended attackers: in local attackers, the attackers used limited resources on specific vehicles, while extended attackers exploit full resources to control several networks.

In particular, in order to provide secure communication in VANETs, a significant knowledge of threats and attacks is necessary to tackle all security challenges. In this section, we discuss the threats and attacks on each security service in VANETs.

#### 4.2.1. Attack on Availability

Availability of information is a very important part of the VANET system, in case a lack of the availability feature may reduce the efficiency of VANETs [73]. In this section, we will explain the threats and attacks on the availability in VANETs.

- Denial of Service (DOS) Attacks: DOS is one of the most common attacks in VANETs, which occur by the internal or external vehicles performing the attacks in the VANET network [18]. The attacker jamming the communication between vehicles and effectively blocking every possible way of action. This attack can be performed by many attackers concurrently in a distributed way, called distributed denial of service (DDoS) [74].
- Jamming Attack: In this attack, the attacker disturbs the communication channel in VANETs by using a heavily powered signal with equivalent frequency [75]. This is the most dangerous attack for safety applications, because it does not follow the valid safety alert. For any successful jamming attack, by performing an action, the jammer can block the useful signal within the same time of the occurrence of an event.
- Malware Attack: The attack can be penetrated into the system through the software components, which are used to operate the OBUs and RSUs [31,65]. If the malware attack occurred in VANETs, that may lead to malfunctioning of the other components of the VANET system.
- Broadcast Tampering Attack: In this attack, untrustworthy vehicles can replicate the same messages by modifying the message or generate and insert a new message information in the VANETs while behaving as a transmit node for inter-vehicle communication [36]. Therefore, this may lead to hide the correct safety messages to dedicated users, which may be the reason for dangerous accidents.
- Blackhole Attack: This is the main attack that targets availability in ad hoc networks, and also exists in VANETs. This attack is usually caused by a registered VANET user. The suspected node receives the packets from the network but it declines the contribution in the networking operation. This may disrupt the routing table and prevents an important message to the recipients due to the malicious node, which pretends to contribute in the non-practical event [1,18,31].
- Grayhole Attack: It is the variant of the blackhole attack. It occurs when untrustworthy vehicles select some of the data packets to forward and dropping the other packet without being tracked [36].
- Greedy Behavior Attack: This attack is normally on the functionality of the message authentication code (MAC), when the malicious vehicle misuses the MAC protocol to increase the large amount of bandwidth, which costs other users. This resulted in overload traffic and causes a collision on the transmission channel, which can produce a delay in the legitimate services of the registered user [76].
- Spamming Attack: In this attack, a numerous amount of spam messages are injected by the attacker such as advertisements in the VANET system, which causes collisions by utilizing more bandwidth [18,65].

#### 4.2.2. Attack on Confidentiality

By using the certificates and by sharing the public keys to all exchange messages, confidentiality guarantees that the messages can be encrypted, where only designated vehicles can get access. Therefore, the vehicles that are outside the nodes cannot understand private and confidential information among the vehicles. Confidentiality is guaranteed through cryptographic solutions. In this section, we will discuss the common threats on confidentiality.

- Eavesdropping Attack: Eavesdropping is very common in wireless communication technology, such as MANETs and VANETs. The aim of this attack is to get the confidential information from the protected data. Therefore, by this attack, secret details can be disclosed with non-registered users, such as stealing user identity and data location, which may be used to track the vehicles.
- Traffic Analysis Attack: This is one of the dangerous attacks that threatens confidentiality. In this attack, after listening to a message transmission, the attacker then analyzes its frequency and tries to extract and gather the maximum useful information.
- Man-in-the-Middle Attack: It can take place in the middle of V2V communication to check closely and alter the messages. The attacker can get the access and control the entire V2V communication, but the communication entities think that they can communicate with each other directly in private [77].
- Social Attack: The social attack is used to divert the attention of the driver. The attacker sends out immoral and unethical messages to the drivers. The aim of attackers is to get the reaction of the drivers after they received this type of immoral messages. Therefore, it affects the driving experience and performance of the vehicle in the VANET system [78].

#### 4.2.3. Attack on Authentication

Authentication is an important part in the VANET system, which is used to protect against the attacks because of the malicious nodes entering the system. The authentication is responsible for protecting VANETs from internal and external attacks [79]. This section highlights the threats and attacks on authentication in VANETs.

- Sybil Attack: It is first discussed by Reference [80]. This is a most dangerous attack in which the node can produce many fake identities to disrupt the normal mode of operations of the VANETs by broadcasting multiple messages using fake IDs. The attacker can manipulate other vehicle behavior, and receive a vehicle system where the messages are transmitted from the different vehicles. Therefore, they feel there is congestion on the road and they are enforced to alter their paths and leave the road clear.
- Tunneling Attack: It is similar to the wormhole attack [18]. The attacker uses the same network to initiate the private conversation, and the attackers join two far-away parts in VANETs by utilizing an extra communication channel named a tunnel. Therefore, the nodes, which are very far-away from each other, can communicate as neighbors.
- GPS Spoofing: In this attack, the attacker uses the trick to create a false GPS location information and did not reveal the correct position to dodge the vehicles that may think it is available in some other location [81].
- Node Impersonation Attack: This attack takes place when an attacker successfully guesses the valid ID of the registered users in the VANETs [31].
- Free-Riding Attack: This attack is very common and is initiated by an active malicious user by making false authentication efforts while being associated with a cooperative message authentication. In this attack, the malicious user may take advantage of other user’s authentication contributions without having its own. This type of behavior is called a free-riding attack. This attack may raise a serious threat to cooperative message authentication [82].
- Replay Attack: It is a very common attack, which is also known as a playback attack. This attack occurs when valid data are fraudulently transmitted or cause a delay to produce an unauthorized and malicious effect. In order to tackle this attack, the VANET must require enough of a time source with larger cache memory, which are used to compare the received messages.
- Key and/or Certificate Replication Attack: This attack is caused by the utilization of duplicate keys and/or certificates of other vehicles as a proof of authentication to create uncertainty, which makes the situation worse for traffic authorities to identity the vehicle. Specifically, the aim of this attack is to create confusion for trusted authorities (TAs) especially in case of any dispute.
- Message Tampering: It is a very common attack, in which the attacker can alter the exchanged messages in V2V or V2I communication.
- Masquerading Attack: The attacker uses false IDs to act as another vehicle. This attack occurs when one user did not show his own identity and pretends to be a different user to legally obtain unauthorized access.

#### 4.2.4. Attack on Data Integrity

The data integrity ensures that the exchanged data do not alter during the transmission. In this section, we will discuss the common threats to data integrity.

- Masquerading Attack: The attacker enters in the VANET system by registered user ID and passwords and tried to broadcast false messages, which appeared to come from the registered node [83].
- Replay Attack: The attacker aims to repeat or delay transmission fraudulently by having a valid data and inject beacon and messages received before the VANET network continuously, which may cause difficulty for traffic authority to identify the vehicles in case of the emergency [84,85].
- Message Tampering Attack: As the name of the attack indicated, this attack normally occurs when the attacker modifies or alters recent message data to be transmitted [86]. For instance, if the route is congested then, the attacker alters the data to clear the road. This can influence the users to alter their driving paths.
- Illusion Attack: This attack receives data from antennas and collects malicious data from sensors, which generate traffic warning messages by using an existing road condition that may create illusion to the vehicles nearby [87]. The illusion attack may be caused by vehicle accidents and traffic congestion and also minimizes the performance of the VANET system by utilizing undesirable bandwidth.

#### 4.2.5. Attack on Non-Repudiation

It ensures that the sender and receiver of messages cannot deny the transmitted and received messages in case of a dispute.

- Repudiation Attack: This attack occurs when an attacker denies to engage in the activity of sending and receiving messages in case of any dispute [35].

## 5. Research Work on Security Services and Privacy-Preserving Authentication

**Figure 5:**
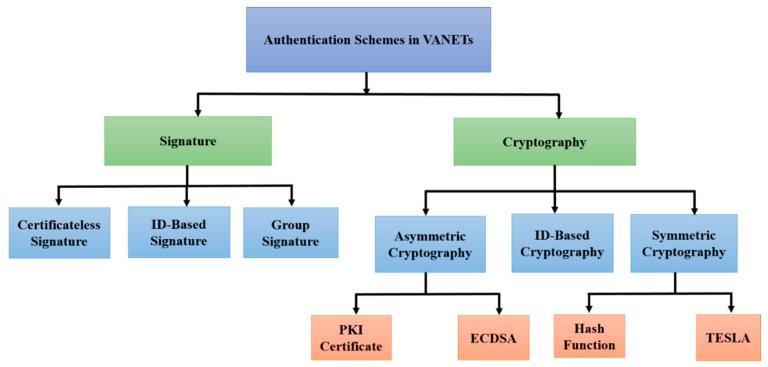
Authentication scheme classification in vehicular ad hoc networks (VANETs).

In vehicular networks, authentication, security, and privacy are leveraged to develop trust among V2V and V2I communications. Utilizing suitable authentication schemes enables a trusted authority to easily identify malicious users and fake messages in order to provide secure communication in VANETs. In this context, several research studies have been proposed to be related to authentication, security, and privacy schemes, which aims to protect VANETs from malicious users, fake messages, and unregistered entities, and tackle all kind of threats and attacks. Several of these schemes utilize cryptography techniques such as symmetric cryptography and asymmetric cryptography to authenticate messages in terms of signing and verifying messages. In this section, we first discuss the recent state-of-the-art methods of VANET security services. Second, we present the authentication schemes in terms of cryptography and signature in detail, as shown in Figure 5.

### 5.1. Research Work on Availability

In recent years, an intense amount of research work has been done, which enhanced the performance of availability services by introducing new protocols. In this section, we will explain the robust existing methods, which have been used to enhance the performance of availability in VANETs.

Kitani et al. [88] presented a new method named as a message ferrying, which is used to improve the message circulation in less populated areas. This method utilized buses to obtain maximum traffic information from vehicles such as location, fuel, and acceleration in their vicinity and then gathered information and forwarded the collected information to the neighboring vehicles. The proposed method was implemented on the NETSTREAM traffic simulator and then compared the information propagation efficiency with other competent methods. In the proposed method, the author did not mention the detail performance parameters, which were involved, and the study was limited to a low density area.

Okamoto and Ishihara [89] introduced a method of information sharing for location-dependent data, which are generated by vehicles using a pull and push method to balance the message delivery and the traffic data assigning the populated area as a message storage area (APAM) scheme. This method is limited to deliver the reliable information based on the pull and push method, but may incur a large amount of computational charges.

Akila and Iswarya [90] introduced an effective data replication technique to manage data access application in VANETs such as location, fuel, and acceleration. Due to a large number of vehicle mobility and change in network topology, which lead to an increase in the parameter changes and cause frequent disconnections. If disconnections happen frequently, then vehicles will not be able to communicate and share data with each other. Specifically, the data replication is used to improve the performance of data access in a distributed system. Since the vehicles contain less storage, they cannot replicate heavy mp4 files or some short duration video clips. This problem is significantly improved by generating the request to the vehicles in a platoon to give some part of their buffers to reproduce data while sharing the same platoon and data among other vehicles. In case a vehicle wants to leave a platoon, it transfers its buffered data to other vehicles prior to leaving a platoon. Therefore, the other vehicles have an access to the data after it leaves. This method has limitations because vehicles frequently leaving and entering a platoon may require a large amount of computational time and incur computational charges.

Park and Lee [91] introduced an effective method to enhance the data accessibility in VANETs by utilizing the data replica of the RSU. In this approach, selection of the data item is made by using the data access pattern and driving pattern, which must be reproduced in the RSUs. Then, the reproduced data are sent directly to the surrounding vehicle without involving communication with RSUs. The main drawback of this approach is, if the data size is larger, then the data replication process may require a large amount of time to handle the replication process. 

### 5.2. Research Work on Confidentiality

In recent years, several methods have been proposed for confidentiality to ensure the safety of data, which contain some useful information from non-registered users in the VANET system. Additionally, it ensured secure communication through a cryptographic solution. In this section, we will explain the existing methods for confidentiality in the VANETs.

Sun et al. [92] introduced a new security system by protecting the confidentiality of sensitive information using shared key encryptions. The aim of the proposed technique is to ensure that the confidential information of the registered users and tracking of vehicles is completed legitimately, which can be done by integrating the new security requirements and design the sophisticated VANET security system against non-authorized users. However, the confidentiality messages are very crucial where vehicles get the useful data from the Internet and RSUs.

Lu et al. [93] introduced a dynamic privacy-preserving key management method referred to as DIKE, which is used to achieve and improve the confidentiality of data in location-based services (LBSs) in the VANET system. In order to control the eavesdropping attack, the confidentiality must be well maintained and protect the service contents from these kinds of attacks. In this method, if a user does not engage in the VANET system, then the user may not join the current VANET system, which does not have access to the current LBS content. To gain the confidentiality in an LBS session, all vehicle users that joined are requested to share a secure session key. That session can be used to encrypt service contents. 

### 5.3. Research Work on Data Integrity

To protect the integrity of the sending message, digital signatures are generated and attached to the messages [94]. In recent years, few studies indicate the accurate and reliable information for preserving data integrity in the VANET system. This section will explain the existing methods for data integrity in the VANETs. 

The main problem occurs when an inaccessible receiving location generates many current packets and forwarding protocols inefficiently in VANETs. To solve this problem, Reference [95] introduced a STAP approach to acquire the receiver’s location privacy preservation in VANETs. By using the concept vehicles, always travel to the busy places and downtown area such as the shopping mall, busy street, etc. In order to achieve data integrity, they deploy RSUs at the main social spot to form a social tier with them. First, the sender computes message authentication code (MAC) and attaches the generated code to the message before sending to the receiver. Once the message is received, in order to obtain integrity, the receiver uses the key session to check MAC. This method is limited to only the busiest place and, due to traffic congestion, this algorithm may consume large memory.

Lin and Li [82] introduced an efficient cooperative authentication technique for the VANETs system. This technique is used to shorten the authentication overhead on individual vehicles and to reduce the delay. To block the various attacks, this method uses a token method to control and manage the authentication workload. When the vehicle passes the RSUs, the vehicle can get the evidence token from trusted authority (TA). Therefore, this token indicates that the vehicle contributed in cooperative authentication before. This method comprises of the large computational algorithm to control the authentication issue.

Lin et al. [96] introduced a group signature and identity-based signature (GSIS) method, which is used to develop secure privacy preserving the protocol based on the group signature and identity-based signature schemes. In case of a dispute, the proposed method protocol can also be used to trace each vehicle, but ID of the sending message need to be disclosed by TA.

### 5.4. Research Work on Non-Repudiation

Li et al. [97] introduced a novel framework with conditional privacy-preservation and repudiation (ACPN) for VANETs. This method utilized public key cryptography (PKC) to obtain non-repudiation of vehicles by ensuring third parties to obtain real identities of vehicles. The identity-based signature (IBS) and ID-based online/offline signature (IBOOS) schemes are utilized for the authentication between V2V and a vehicle to road side unit (V2R). This method significantly reduced the computational cost. However, the handling of managing certificates is complex due to IBS and ID-based online/offline (IBOOS) authentication schemes.

### 5.5. Requirements of Autheticaion

In VANETs, authentication can be done by two ways. First, at the level of the node, called node authentication. Second, at the level of the message level, it is called message authentication. Verifying the message integrity plays an important role for improving the VANET security system. Therefore, message authentication was regarded as a key parameter in the VANETs [98].

In order to provide secure communication in VANETs, there are some requirements of authentication, which must be satisfied, are listed below.

- Computational and Communication Overhead: The number of cryptographic operations performed by a vehicle or trusted authority for verifying an authentication request must be minimized. For example, the time required to process a digital signature in authentication must be controlled.
- Utilization of Bandwidth: The bandwidth utilization is very important in the authentication and must be utilized properly in bytes per second (bps) to handle a request for an authentication such as exchanging cryptographic secret keys and credentials.
- Scalability: The process of the authentication is scalable, which can handle multiple network operations and communications.
- Time Response: The time that is needed to respond for an authentication mechanism must be reduced.
- Powerful Authentication: The authentication schemes must have a good capability to prevent VANETs from attacks.

### 5.6. Privacy-Preserving Authentication

Privacy means everyone has the right to keep the information confidential and decide whether or not to share any information with others. Specifically, privacy is a system that is used to protect the sensitive and confidential information of the vehicles or passengers from the attackers. The privacy of vehicles should be considered an important issue in the VANET system. Lately, an intense amount of research studies have been done in security and privacy services in the VANET system, which ensures vehicle safety and improves the traffic flow. Anonymous authentication is one of the most well-known approaches. Most of the recent existing works are relying on a pseudonym-based approach, which can be used to protect the privacy and security of the vehicle users. By utilizing the pseudonym-based approaches, users can get better and more robust privacy preservation. To control privacy attacks, the trusted authority need to change pseudonyms frequently. The privacy is further categorized into two types: (i) privacy of user and (ii) user location privacy.

- Privacy of User Protection: It is used to prevent the personal information of users from malicious users or attackers.
- User Location Protection: Meant to protect user’s information such as vehicle location at a certain time and route information.

The aim of authentication schemes is to identify the malicious nodes and bogus messages. Thus, it provides security in VANETs. Regarding authentication schemes, several research studies have been proposed, which ensure the secured communication in VANETs. These schemes utilized different cryptography techniques for signing and verifying the messages. In this paper, we discussed the authentication schemes in terms of cryptography and signature in detail, as shown in Figure 5. Cryptograph-based authentication schemes are categorized into symmetric cryptography (Hash Function and timed efficient stream loss-tolerant authentication (TESLA)), asymmetric cryptography (PKI certificate and ECDSA), and identity-based cryptography. Signature-based authentication schemes are separated into identity-based signature, certificateless signature, and group signature. The classification of authentication schemes in each category are discussed in terms of analysis and their related works.

#### 5.6.1. Symmetric Cryptography Scheme

This authentication-based cryptography is also called private-key cryptography. This scheme utilizes a message authentication code (MAC) to authenticate the messages. By using a shared secret key, sender can generate MAC for each message, and also all nodes in an anonymity set verify the MAC attached with the messages by using that key. Symmetric cryptography is fastest and obtains robust computational efficiency because of a single key. To achieve a high level of reliability and privacy, Choi et al. [99] introduced a new method, which can produce high efficiency of privacy by combing symmetric authentication with the short pseudonyms in VANETs. In this method, to generate short-lived pseudonyms, authority need to send the different ID and seed value to each vehicle. Additionally, RSU is capable of verification for MACs because it can share keys with vehicles. 

Xi et al. [100] proposed a random key-set based authentication to maintain user privacy by using a zero-trust policy without having trust of central authority with a user policy. In this method, the anonymity is improved by using independent keys for authentication at neighboring RSUs, and also identifying attackers and key revocation, which have been considered in terms of a practical application. This method is better but requires a large amount of computational cost to handle a zero-trust policy. As we know that, symmetric cryptography-based authentication consists of two major issues. First, the key management system in VANETs is very weak, which can elevate the communication overhead and storage. Second, this technique has a lack of non-repudiation. Therefore, it is difficult to provide authentication to each vehicle.

Vijaykumar et al. [101] designed a TA to facilitate online services to the customers via VANETs. Therefore, it is vital that the exchanged communication between the TA and VANETs preserving the confidentiality and authentication of messages. Besides that, a dual authentication and key management technique are used to provide a secure transmission of data in the vehicular network. A dual authentication technique offers sufficient security to the vehicle, which can efficiently intercept the malicious vehicles to enter in the VANETs.

Lin et al. [102] introduced a time-efficient and secure vehicular (TSVC) method with privacy preservation. This method is used to significantly reduce the packet consumption without limiting the security requirements. The authentication of the packet is done by a MAC tag, which is attached to each packet. However, it required a fast hash operation to verify each packet. By using this method, the packet overhead is minimized by reducing the signature overhead and its verification latency. The bandwidth utilization decreases with the decline in the number of packets. Rhim et al. [103] introduced an efficient method for a MAC-based message authentication scheme, but this method cannot tackle and secure against the replay attack and requires a sophisticated algorithm, which was proposed by Taeho et al. [104] by utilizing an improved MAC authentication scheme for the VANETs’ system. Zhang et al. [105] presented a new method to authenticate a message by using the roadside unit aided message authentication (RAISE) technique. In this approach, RSU verifies the authentication of messages, which are transmitted from vehicles to notify the results back to vehicles. Instead of verifying the message through a traditional PKI-based scheme, they used the concept of each safety message attached with a MAC. This was generated by the sender by using the secret key and RSU. Then RSU is responsible to verify MACs and circulate the outcomes of message authenticity to other vehicles within their range.

##### Hash Function-Based Authentication Schemes 

The next category of symmetric cryptography is a hash function, which is responsible to examine the message integrity without any encryption of the message. The message is an input in the hash function, which can generate a fixed string referred to as the hash value. In order to ensure the message integrity, the hash value must be attached with a sending message. Chuang et al. [106] introduced a decentralized-based lightweight authentication scheme named as TEAM (trust-extended authentication mechanism). This technique uses the concept of transitive trust relation, but the amount of cryptographic in TEAM is less than compared with other existing methods because it only utilizes XOR and the hash function during the authentication process.

Chim et al. [107] introduced a method, which discussed the security and privacy issues of V2V in VANETs. This scheme utilizes one-way hash function and a secret key between vehicle and RSU. Therefore, this methodology can resolve the privacy issue, which may occur during communication. Vighnesh et al. [108] introduced a novel sender authentication technique for enhancing VANET security by using hash chaining and the authentication code to authenticate the vehicle. This method ensures that the secure communication between the vehicle and RSU. Confidential data is encrypted through the master key. Before sending packets to the authentication center, the RSU attaches its identity, which can eliminate the possibility of rogue RSU abusing the VANET network. He and Wen [109] presented a method that addresses the problem of the DOS attack against signature-based authentication. To tackle the DOS attack, the pre-authentication can be done before signature verification. In this method, the pre-authentication using the one-way hash chain cannot be classified as a full authentication scheme. 

##### TESLA-Based Authentication Schemes 

The symmetric cryptography is further extended to time the efficient stream loss-tolerant authentication (TESLA). In this approach, first, the sender computes MAC using a known key and attaches a MAC to each sending message, and the receiving messages are buffered without authentication at the receiving part. The main disadvantage of TESLA is that the advance synchronization of the clock at receiving side is required with the clock at the sending side. Additionally, TESLA is vulnerable to the DOS attack in terms of memory, which is caused by unregistered vehicles that utilize receiver memory with fake messages [37,110].

Jahanian et al. [111] introduced a TESLA-based technique. In this technique, the timed method checking the approach based on the timed color Petri model is used to design and verify TESLA. Lately, the researchers have found that the two factors that need to be analyzed first include security efficiency and the percentage of successful attack. Studer et al. [112] introduced a modified form of TESLA, which is known as TESLA++, and it provides the same broadcast authentication, which is computationally efficient since TESLA with less memory consumption presented a method to effectively verify the newly RSUs and OBUs, which were encountered during communication. The goal of TESLA++ is to control memory DOS attacks, which can be obtained by receivers’ self-generated MAC. This may be lower down the memory requirements for authentication. However, TESLA does not offer multiple hops authentication and non-repudiation [37].

#### 5.6.2. Asymmetric Cryptography Scheme

The next authentication scheme in VANETs is referred to as asymmetric cryptography, which is also called public key cryptography. This technique can be used for encrypting and decrypting a message to ensure the security of data in the major communication network. Specifically, the asymmetry can be used to encrypt a message, which can be done either by using a public key or by generating a digital signature. Normally, a private key is only used for decrypting an encrypted message and for verifying a digitally-signed message. 

Asymmetric cryptography is further classified as a public key infrastructure (PKI) certificate and elliptic curve digital signature (ECDSA) based authentication.

##### Public Key Infrastructure Certificate Schemes

Most of the vehicles contain public or private key for pseudonymous communication. In order to achieve a secure and reliable way, the public key certificates are the best methods that are used in public key infrastructure (PKI) to authenticate vehicles. It contains the digital signature of the certification authority (CA) and vehicle key for authentication [44]. The CA is the centralized management unit, which is responsible to certify nodes, keys, etc. Furthermore, it can also authenticate the vehicles in V2V communication. Every vehicle needs to be register with the CA database before officially join the VANET system. The vehicle can communicate with CA in two ways either directly, such as offline registration or via RSU as an indirect online registration. Raya et al. [113] introduced a new method, which utilizes the anonymous public keys to provide privacy. The anonymous keys must be changed in the way that the receiver will not be able to track the vehicle owner key. The main demerits are that it required a large amount of storage and memory and also requires a huge amount of certificate revocation list (CRL) checks, since using a large number of anonymous keys. Therefore, it may be the reason for the DOS attack due to a large amount of computational overhead.

Calandriello et al. [114] introduced a robust pseudonym-based authentication method to reduce the security overhead but keeps maintaining the robustness of traffic safety. This scheme alleviates the limitations of a pseudonym by using the combination of the baseline pseudonym and group signature, which can generate its own pseudonym on-the-fly and self-certification [37]. Additionally, it minimizes the requirements of handling the pseudonym in authentication. In Reference [115], Wasef and Shen introduced an expedite message authentication protocol (EMAP) method, which adopts public key infrastructure (PKI) and certificate revocation lists (CRLs) for their security. In this scheme, EMAP for VANETs replaced the lengthy process of CRL by an effective revocation process. This process uses a keyed hash message authentication code (HMAC) in EMAP. The purpose of the key is used to calculate HMAC and is shared only among non-revoked OBUs to safely share and update the secret key. Due to the message verification delay, which resulted in a significantly reduced message loss ratio in EMAP, as compared with the conventional authentication.

Reference [116] proposed a new scheme in which vehicles generate a request to CAs for short-term pseudonyms during specific intervals. To reduce the communication overhead with CAs, Reference [117] introduced the self-issuance scheme to enable the vehicle to generate pseudonyms independently. Rongxing et al. [118] introduced a new method for effective pseudonyms changing at the social spot (PCS) for privacy of location, by determining several vehicles gathered on specific spots such as an intersection or the parking area. It utilizes anonymity size as a privacy metric (ASS) and, if ASS reaches the threshold, then the pseudonyms are changed simultaneously. However, it cannot perform well in a low density.

In the year 2010, Schuab et al. [119] proposed a new approach that does not depend on the pseudonyms-identity mapping to achieve accountability but instead, resolution information is embed with V-token pseudonym certificates. In this scheme, by using the V-token approach, each vehicle carries its own resolution information, which can provide a scalability advantage. The main challenge is the revocation of the pseudonym certificate, which may limit the scalability in the VANET. In case, if the vehicle long-term certificate is revoked, then the vehicle cannot obtain a new pseudonym from CAs. In recent years, few studies have been proposed on the CRLs’ distribution methods [120,121]. These methods are unable to stop the revoked vehicle from continuously communicating in VANETs until and unless all the pseudonyms become inactive. The drawbacks of checking the CRL process make it unreliable to authenticate a large number of messages under the specific period in VANETs [122].

Azees et al. [123] introduced an effective anonymous authentication with a conditional privacy (EAAP) scheme to avoid a malicious vehicle from entering in the VANETs. This scheme is used to track mechanism and track vehicles or RSUs that create disturbance to VANETs. Recently, bilinear pairing was introduced in which the TA in EAAP is not required to keep the anonymous certificate of the vehicles and RSUs [124]. Additionally, the TA has the right to cancel the anonymity of a disobedient vehicle and reveal their identity in a group. Then, the revoked identity added to the identity revocation list (IRL) was managed under the supervision of TA.

##### Elliptic Curve Digital Signature (ECDSA) Based Authentication Schemes

The next part of asymmetric cryptography is an ECDSA authentication scheme, which is an analogue type of digital signature dependent on the elliptical curve cryptography [125]. Manvi et al. [126] introduced an ECDSA-based message authentication scheme in VANET. This technique utilizes a secure hash algorithm (SHA) by the sending vehicle to generate a private and public key, and also create a hash of the message by using SHA. At the receiving part, the received message is decrypted by using the public key. Kalkundri et al. [127] presented a new technique, which utilizes the ECDSA algorithm to obtain message authentication. Furthermore, it can also provide security in terms of the point-to-point (p2p) mechanism to obtain authentication in VANETs. The combination of p2p and ECDSA along with VANETs can improve the efficiency of the algorithm and also minimizes the message delay. Smitha et al. [128] proposed a new method, a classification of the critical safety message, and provided an adaptive way to authenticate the message based on the Merkle tree and ECDSA. This scheme discussed the DOS, man in the middle, and phishing attacks. Furthermore, this approach can increase the message authentication delay.

#### 5.6.3. Identity-Based Cryptography Schemes

The identity-based cryptography (IBC) is similar to the asymmetric cryptography, in which the user public key can be obtained from their ID information such as user location, telephone number, email address, and more. In order to authenticate the message, the IBC authentication schemes did not utilize PKI certificates. Thus, it significantly reduced the communication overhead and managed overhead of CRLs.

In Reference [129], Shim proposed an efficient conditional privacy-preserving authentication (CPAS) scheme. This scheme utilized pseudo-IBS in order to obtain secure communication in V2I. In this scheme, the RSU is able to verify the large amount of received messages simultaneously, which significantly reduced the large amount of computational time and also reduced the memory space. In Reference [92], Sun et al. proposed a VANET security system to obtain the vehicle’s privacy by utilizing an identity-based cryptography system. In this scheme, an authentication is accomplished without needing a certificate. Since the proposed scheme did not uses certificates, it consumes less memory space, and the message and computational overheads are reduced, as compared to other methods. In Reference [130], Bhavesh et al. introduced an authentication with multiple levels of the anonymity (AMLA) protocol by utilizing pseudonyms and identity-based signature (IBS) scheme. In this approach, the AMLA protocol significantly reduced the message overhead as compared to other PKI methods. Li et al. [97] proposed a public key cryptography technique to generate the pseudonym. In this scheme, the identity-based signature (IBS) and ID-based online/offline signature (IBOOS) schemes are utilized for the authentication between V2V and vehicle to road side unit (V2R). These schemes provide authentication, privacy-preservation, and non-repudiation. However, the handling of managing certificates is complex in IBS and IBOOS authentication schemes. 

#### 5.6.4. Identity-Based Signature Schemes

The identity-based signature (IBS) uses node identifiers in terms of the public key and sign messages with the private key generated from the identifiers [131]. In IBS, the private key generator (PKG) is used as a trusted authority for generating and assigning the private key. Recently, new identity-based signcryption (IBSC) has been introduced by utilizing bilinear pairing, which required strict analysis of security based on robust security modeling without considering a random oracle background, which can indicate that the IBSC is considered a reliable method [132].

The IBS has the four-step process: setup, key extraction, signature signing, and verification, as follows.

- Setup: PKG evaluates the master key and public parameters. Then, PKG discloses these parameters to all vehicles publicly in the VANET.
- Key Extraction: PKG uses the vehicles ID and master ID to compute a private key. Then the PKG sends these private keys to communicate with the vehicle through a secure channel.
- Signing Signature: Signature SIG can be generated by using a private key by assuming a message M, and timestamp T.
- Verification: This algorithm is used to find out whether signature SIG is valid or not by having these parameters such as identity, signature, and message.

To overcome the computational overhead in the IBS scheme for VANETs, Lu et al. [133] proposed a novel authentication framework with adaptive privacy using an ID-based online/offline authentication (IBOOS) technique. In Reference [134], Zhang et al. introduced an identity-based technique for signature hierarchical aggregation and batch verification. In this approach, identity-based signatures are generated from different vehicles, which can be aggregated and verified in a batch, and the message collector is used to re-aggregate the aggregated signatures. This scheme significantly reduced the large computational process of certification verification by utilizing the identity-based vehicles and RSUs. In 2001, a new hash-sign-switch method has been proposed based on the trapdoor hash function. In this scheme, the signing process is efficiently divided into the online and offline phases. Due to a good pairing process, the efficiency of this method is better than the IBS scheme. However, the requirement of memory storage space makes IBOOS unsuitable for VANETs [135]. In the verification, the IBS eliminates the requirement of certificates, which can be used in the verifications of public keys. Thus, it did not need to be distributed as public keys, which are related to certificates [44]. Specifically, only PKG knows the private keys in VANETs because it is generated by PKG in IBS, which may generate an escrow problem. To overcome this problem, in the year of 2017, Zhang et al. [136] introduced an efficient technique to authenticate the vehicle protocol named as a distributed aggregate privacy-preserving authentication (DAPPA), which is based on multiple trusted authorities with the IBS technique.

In Reference [137], Zhang introduced a novel approach based on a new security tool named as one-time identify-based authenticated asymmetric group key to develop cryptography mix-zone (CMIX) against eavesdropping. In 2018, Zhang et al. [138] introduced a secure privacy-preserving communication scheme for establishing vehicle cloud (VC) and data broadcasting in the VC. In this approach, a group of vehicle, which are located near VANETs, are used to develop a secure and dynamic VC. Therefore, it enables all the vehicle resources to be integrated and exchanged data securely, and any cloud user can process their data securely once the VC is formed.

**Figure 6:**
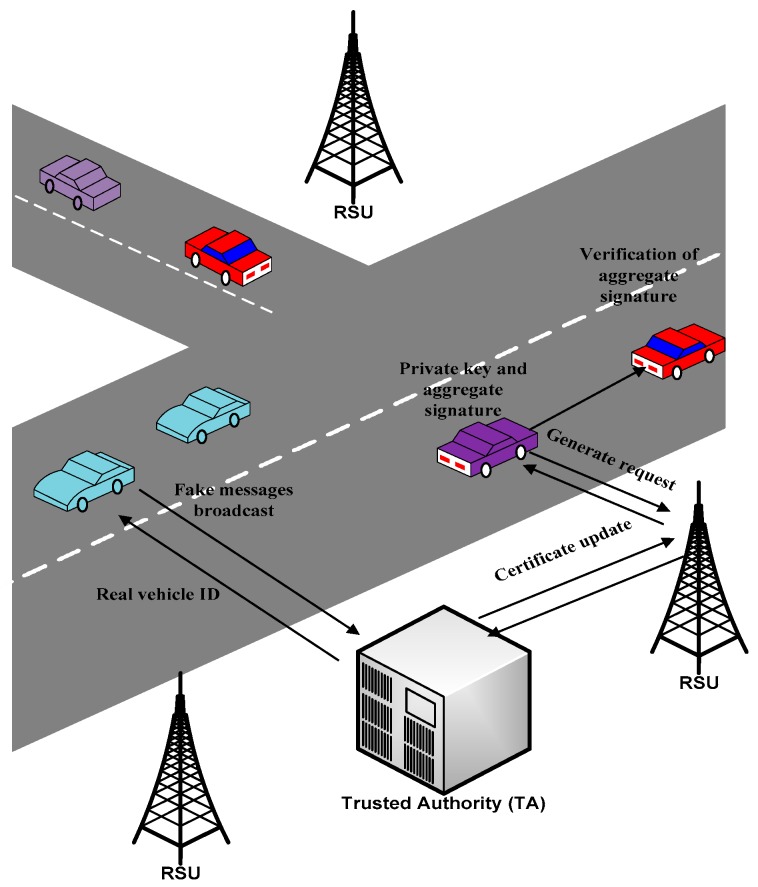
Graphical representation of distributed aggregate privacy-preserving authentication (DAPPA).

Figure 6 illustrates the diagram of DAPPA, in which the RSU consists of a large communication range, as compared with the vehicles. Each RSU consists of an initial key pair and a corresponding certificate, which is issued by the trusted authority [44]. Every vehicle contains the secret to develop secure channels with RSUs, and every vehicle generates a request to RSUs when entering into the communication range to share its private key. The vehicle utilizes the shares for each message to generate a one-time private key and a one-time ID-based signature within the authorization period. Then, the other users can utilize the sender’s ID to verify the signature. By using a one-time private key, distributed aggregate privacy-preserving authentication (DAPPA) resolves the escrow problem, which is not rooted by the trusted authority. However, it can make the system more complex since the vehicles need to request the shares from an adjacent RSU. Furthermore, the utilization of the one-time private key and ID-based signature can cause the delay and significantly reduces the communication efficiency in the VANET system [44].

#### 5.6.5. Certificateless Signature Schemes

The certificateless signature is used to overcome the high cost of certificates based in the PKI technique to resolve the escrow issue in IBS. In 2003, the certificateless public key mechanism is presented for the first time [139]. In the certificateless cryptography, the key generation center (KCG) plays an important part, which works as a third party and is responsible for providing the user with a private partial key DIDi, which is evaluated from the identity of user IDi. 

The secret value the user can generate include the actual private key and the partial private key delivered by a key generation center (KGC). In contrast to the ID-based cryptography scheme, the KGC may not have access with this type of private key. Consequently, a user can use secret values and different parameters to produce his public key identity known as PKIDi. The certificateless signature (CLS) method is categorized into seven different algorithms, such as setup, partial private key extract, set secret value, set private key, set public key, sign, and verify [139]. All these algorithms are discussed below.

- Setup: Setup utilizes a security parameter k to produce the master key msk and master public key mpk. Furthermore, it can also produce parameters param, which can be distributed among all nodes [44].
- Partial Private Key Extract: It can produce a partial private key D_ID_ by having different parameters such as master key, master public key, system parameters, and an identity ID [44].
- Set Secret value: The secret value x_ID_ is generated by using the master public key and other system parameters param [44].
- Set Private Key: This algorithm utilizes param, partial private key D_ID_, and secret value x_ID_ as input parameters. The secret value x_ID_ is used to transform D_ID_ into full private key P_A_. The algorithm returns P_A_ [139].
- Set Public Key: It generates the public key PK_ID_ by using different parameters such as the master key, the system function, an identity, and its secret value [44].
- Sign: It generates a certificateless signature by using a system parameter, a master public key, and more [44].
- Verify: It can verify the signature by using several parameters such as the system parameter, master public key, ID, the ID public key, and more [44].

The security models are further classified into two types such as super type I adversary A_I_ and super type II adversary A_I I_ [140]. A_I_ solves the real-world adversary who can obtain IDs and some valid signatures, while A_I I_ solve the malicious KGC who contain the master secret key and can initiate the attack such as the eavesdropping attack on signatures and create signing queries. In recent years, there are few research studies that have been proposed regarding certificateless signature referred to as a certificateless short signature (CLSS) [141,142]. These schemes proved to be the secure method against A_I_ and A_I I_ in the random oracle model [44]. At the end of 2014, several researchers have proposed the CLS scheme without utilizing any pairing to increase the efficiency, but the signature length is very large, which cannot constrain unlimited bandwidth and storage devices in VANETs [143,144]. In 2015, a scheme for V2I communication based on a certificateless signature is introduced. In this technique, conditional privacy preservation is obtained by mapping the traffic message transmitted by the vehicle into false identity. In case of dispute, the responsible authority can recover the real identity from the pseudo identities [145]. Furthermore, this method produces efficient computational overhead in comparison with other competent techniques. At the beginning of 2018, Cui et al. [146] introduced a method for a certificateless aggregate signature based on an elliptic curve cryptosystem (ECC), which can support conditional privacy preservation. It provides secure communication between V2I in VANET. Furthermore, it can satisfy privacy requirements and also achieves lower message overhead, which are advantages over other methods.

#### 5.6.6. Group Signature Schemes

The vehicle privacy is preserved in the group signature based schemes, which can allow registered members of the group to sign up for the messages anonymously as a representative of the group [19]. The head of the group has the right to find out which sign is coming from the original sender. The group signature usually requires a large amount of time to verify the signature, which makes it limited to time-related applications in VANETs.

In 2010, Zhang et al. [147] proposed an efficient method that utilized each RSU to maintain and managed on-the-fly group within its range of communication, and the vehicles, which enter the group and can secretly send V2V messages. These can be further verified by users of the same group, if any bogus message produced by the vehicle can be traced by the trusted authority. In 2011, Park et al. [148] presented an RSU-based decentralized key management (RDKM), which is used only for multicast services in vehicle communication (VC) systems. This scheme reduced the large amount of rekeying overhead by contributing some portion of the key management functions to the RSUs and also by updating the key encryption keys (KEKs) within the RSUs.

To alleviate the overhead of revocation, the distributed management system is a promising approach. Based on this technique in 2012, Sun et al. [149] introduced a distributed key management system (DKM) in the VANET system. In this scheme, the domain is formed into small sub-regions, and the vehicle needed to update it is the secret key from the regional head of the group, which manages the region. In this approach, during the updating process, the DKM restricts the vehicle to disclose the updated value of a secret key to the regional head of the group. However, the anonymity feature of the group signature makes it vulnerable to attack by a malicious user through the broadcasting of fake messages. Malina et al. [150] introduced a group signature with short-term linkability and categorized the batch verification. This method produced efficient signing and verification as compared to other competent methods. In Reference [151], Zhang et al. introduced a location based service (LBS) protocol, which is used to address the inherent challenges in terms of authentication and conditional privacy for offering LBSs in VANETs. In this scheme, the providers of RSUs and LBS are identity-based, and a vehicle only requires a member key. By using this key vehicle, it can generate verifier-location group signatures. The LBS validates these signatures without interfering the privacy of a vehicle. If an LBS request is found to be false, then the key generation certificate can evaluate the vehicle ID.

Islam et al. [152] introduced a password-based conditional privacy preserving authentication and group-key generation (PW-CPPA-GKA) protocol for the VANET system. This method provides several features such as user exiting, user entering, and changing a password. This protocol is computationally stable since it is designed without using bilinear pairing and elliptic curve techniques.

## 6. Simulation Tools

When applications are designed and developed for VANETs, privacy and security should be considered seriously. The main problem occurs when evaluating the performance of security and privacy due to the limited features of the VANET system in terms of mobility, network structure, and decentralization. To get the optimal solution, it is important to design and develop sophisticated simulation tools that can be used to produce the VANETs results efficiently. VANET simulation tools are categorized such as the mobility simulator and network simulators. Specifically, the mobility simulator is used to generate vehicle mobility [44]. The network simulator is mainly used for evaluation and performance of the VANETs and also indicates the issues related to the network.

### 6.1. Mobility Simulator 

In the VANET system, the mobility model can determine the movements of the node, which are linked with the simulator. By using this terminology, the simulator generates random topology based on each vehicles’ condition [32]. The mobility model consists of two patterns named traffic and the motion patterns, respectively. The motion pattern is determined by the behavior and attitude of drivers, which can create vehicle movements with pedestrians and vehicles [32]. The traffic generator produces random topologies and a map, which are used to evaluate the vehicle behavior, according to the traffic environment. Generally speaking, it is a major challenge to manage system modeling to integrate with the real traffic environment. Therefore, designing the mobility model can be done by using several other types of models depending upon the traffic conditions and situations. The mobility model can be categorized into two types such as macroscopic and microscopic models [44].

The METACOR [153] utilizes traffic at high scale and is also used to determine the vehicle attitude. The METACOR is very useful for providing the macro of the traffic environment.

VanetMobiSim [154] is the generator of realistic vehicle movement traces for networks simulator. It reviews and validates the microscopic and macroscopic mobility description and adds details to them, and can reproduce the general vehicular traffic environment.

Simulation of urban mobility (SUMO) [155] is an open source micro-traffic simulator that can generate the vehicle traffic and update the vehicles parameters such as speed and positions. It is a microscopic traffic simulation that can import city maps with a different file format and version. Each vehicle contains user ID, time of departure vehicle, vehicle routes and location, etc. [155]. Additionally, SUMO is capable of handling highly integrated simulations that can be used for large networks, and it is able to get timely feedback from the network simulator. Specifically, SUMO is more reliable and suitable for V2X communication by considering an individual vehicle behavior and feedback of each vehicle by updating the network simulator for future processing.

### 6.2. Network Simulator 

The University of California at Berkley and the VINT project jointly developed the NS-2, which is a discrete simulator developed for networking research. Recently, many network simulators such as NS-2 [156], NS-3 [157], GlomoSim [158], and OMNeT++ [159] are used to evaluate the performance of the model and measure the privacy and security of routing protocols in VANETs. Additionally, many programming languages such as C++ and Java are used to construct simulators.

The NS-2 simulator is designed and written in C++ with an object tool command language (OTCL). NS-2 is mainly used for research in network communication to support simulation for routing, and the multicast protocol through wired networks [156]. The main disadvantage of NS-2 is that the node must be programmed manually by the users in order to find the vehicle in their vicinity and establish communication. To overcome this problem, NS-3 provides an optimal solution, which is used to improve the network modeling and reliability. NS-3 can also provide an interface for Python and mechanism to integrate with the other open source platform [157].

The global mobile information system (GlomoSim) was developed in California, USA, which is used to simulate the wireless network. It is one of the most famous techniques of the network simulator after NS-2. GlomoSim is capable of running on a shared-memory symmetric processor (SMP) and assist in dividing the network into separate modules and each functioning with different processes [32]. The main purpose of GlomoSim is to support millions of nodes performed as a single simulation. Aggregation of nodes and the layer are the limitations of most network simulators.

The OMNeT++ is the discrete simulation library, which was developed to simulate the network communication, multiprocessing, system configurations, and other distributed systems [159]. Specifically, OMNET++ provides a simulation platform, which can be used to design simulation modeling, and also provide more reliability for the larger mobility of VANET applications [44]. 

### 6.3. Comprehensive Simulation

To change traffic settings, the information that is received from the network simulator must be processed by the mobility simulator. SUMO is used to generate high mobility traffic to simulate the vehicular network because of its unique characteristics of network traffic. SUMO has a function that can simulate a single part and whole cities in one simulation [160]. 

The traffic and network simulator environment (TraNS) is a Java-based visualizing tool consisting of SUMO and NS-2, which is specially designed for the VANET system. A new TraNs Lite version is developed for the mobility generator, which excluded the NS-2 network simulator [32]. The major disadvantage of the TraNS simulator is that it cannot support a highly large-scale network and is also not cost-efficient in terms of acquiring the protocol of VANETs [161].

An integrated wireless and traffic platform for a real-time road traffic management solution (iTETRIS) [162] aims to enhance the large-scale simulation network of VANETs for evaluating the services for transport and traffic management. In order to get the real-time closed-loop coupling simulation platform, the iTETRIS combined with SUMO and NS-3, which can be considered an extension of TraNS [161]. 

The vehicles in network simulation (Veins) is an open-source framework [163], which is used to run vehicular network simulations. Veins implement the IEEE 802.11p protocol at the physical and the MAC layers and is responsible to manage the data transfer between OMNET++ and SUMO through TraCI [164]. The main advantage of bi-directional coupling has two features. First, the network simulation mainly controls the mobility of simulation for handling the traffic communication in VANETs. Second, information that includes the position or routes could be provided by mobility simulation to the network simulation. Additionally, the veins offer a comprehensive function to achieve bi-directional coupling, which can provide better accuracy for the development of protocols [163].

### 6.4. Performance of Authentication Schemes

In order to evaluate which algorithm of authentication schemes obtain better results in each category. Several papers have been reviewed related to the authentication schemes. In symmetric cryptography, Reference [101] obtained a better result by using a JAVA-based simulator, in which, considering multiple nodes, each node acts as a VANET user. Additionally, the proposed dual key management scheme significantly obtained computationally efficient and secure data transmission. Thus, in symmetric cryptography, this scheme obtains better results as compared to the other competent methods.

In asymmetric cryptography, Azees et al. [123] introduced the EAAP scheme to avoid malicious users to enter in VANETs. In this approach, Cygwin 1.7.35-15 with the gcc version 4.9.2 was used to evaluate the computational performance of EAAP. The proposed scheme achieved low computational cost by verifying the multiple signatures and certificates in 300 ms as compared to the other competent methods in an asymmetric cryptography scheme.

In identity-based signatures, Zhang et al. [134] introduced a privacy-preservation scheme based on an identity-based signature. The proposed method used NS-2 [156], VanetMobisim [154], and the cryptography library MIRACL [165] to evaluate the performance by assuming different degree parameters, and generating the vehicle mobility with the speed ranges from 50 to 60 km/h. This scheme was significantly used to reduce the transmission overhead, and also minimize the vehicle waiting time to initiate the batch verification process. Therefore, this scheme is the best scheme among other competent methods.

In a certificateless signature, Cui et al. [146] introduced a certificateless signature based on ECC, which can be able to support conditional privacy preservation. Several different parameters have been used to evaluate the performance of this scheme. First, we created a bilinear pairing with a security level of 80 bits. In the system model, two different layers of the vehicular network are considered. The first layer consisted of a vehicle and RSU, and the communication medium between OBUs and RSUs is 5.9 GHz DSRC. The second layer consists of TA and KGC. In this approach, each vehicle transmits a traffic related message in 300 ms, and the total time need to verify more than 650 signatures is less than 300 ms. Thus, it can verify the large number of messages simultaneously. In the proposed method, the message overhead is significantly reduced along with the less computational and transmission cost as compared to the other certificateless signature schemes.

For the group signature scheme, Zhang et al. [151] introduced a location-based service (LBS) protocol. In order to evaluate the efficiency of the proposed method, this scheme considered LBS and revocation stages. In the simulation, the cryptography tool multiprecision integer and rational arithmetic library (MIRACL) [165] and MNT curve implement on embedded degree, which is built in C language, are used. In each LBS event, the RSU processes only one pair to decrypt the message, which required 2.17 ms. This approach can protect the identity and privacy of the vehicle by providing the service to the vehicle anonymously. Thus, it can outperform other competent methods.

## 7. VANET Applications

VANETs provide a communication to nearby vehicles by V2V and vehicles to other communication devices such as V2I, V2R, and more. In particular, V2V and V2I communications offer the development of various applications and provide a wide-range of safety and comfort information to the drivers and passengers. Vehicles are equipped with GPS receivers, network devices, and electronic sensors to process and collect traffic-related information and then transfer the information to the other vehicles and infrastructures. These exchange information between V2V and V2I enhance the traffic and road safeties, by improving driving experiences and passenger safety [62].

**Figure 7:**
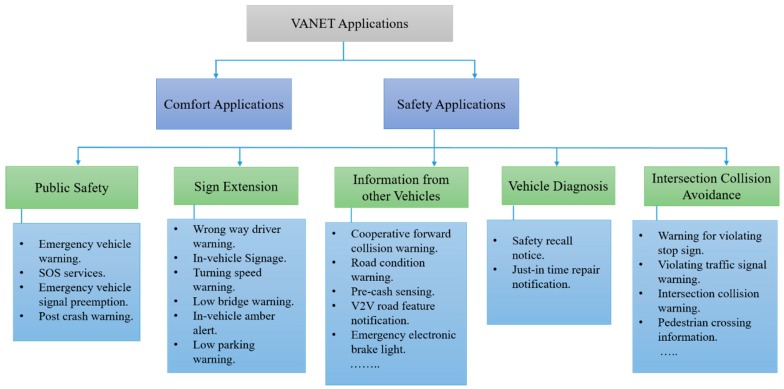
VANET applications.

VANET applications are classified into two categories: (1) comfort applications, and (2) safety applications, as shown in Figure 7.

### 7.1. Comfort Applications

The comfort applications are referred to as non-safety applications, which aim to enhance driver and passenger comfort. These provide the drivers and passengers with updated climate information, Internet access within the network range, communication exchange between vehicles, detail the location of hotels, and nearby restaurants and patrol stations [62].

### 7.2. Safety Applications

The safety applications of VANETs are used to enhance the protection of all users of the road networks. In these applications, vehicle-to-vehicle and/or vehicle-to-infrastructure communications can be used to improve the traffic and road safeties, lane changing warning, emergency video streaming, and by avoiding collisions and accidents [62]. 

Safety applications have requirements to collect traffic information from a vehicle sensor and then process and broadcast this information in the form of the message to the other vehicles and infrastructures. In ITS, vehicles are equipped with wireless communication technology in order to communicate and exchange information with other vehicles and infrastructures, i.e., V2V and V2I. From Figure 7, we observe that the safety applications utilizing V2V and/or V2I communications can be categorized as below [166].

#### 7.2.1. Public Safety

When an incident has occurred, public safety applications provide support to the drivers, and help emergency response teams by reducing their travel times and providing emergency services to them. In particular, the average emergency vehicle response time is approximately six to seven minutes. Public safety applications utilize the frequency of 1 Hz for both V2V and V2I communications with a communication range that varies from 300 to 1000 m [167]. This application can be further categorized as shown below.

- Emergency Vehicle Warning: The emergency vehicle warning system is used to guide the driver and provide a clear road to the emergency vehicles such as ambulances, fire brigade vehicles, etc. in order to reach their destinations without being stuck in the traffic. This application is implemented by broadcasting the safety or alert messages relying on V2V communication to clear the road for an emergency vehicle. The broadcast message has an information of emergency vehicle speed, direction, route, etc. [32].
- SOS Services: This system is applicable in life threatening and fatal accident conditions by transmitting SOS messages. The SOS signal is activated either automatically or manually. This service is applicable for both V2V and V2I communications.
- Emergency Vehicle Signal Preemption: Traffic infrastructure at each intersection is used to support emergency vehicles by broadcasting messages to all traffic lights en route to the destination by using V2I communication. In case of an emergency, the vehicle arrives at a signal and all the lights are green to pass the emergency vehicle, which reduces the response time for the emergency vehicle, which then subsequently reduces the chances of an accident [32].
- Post-Crash Warning: A post-crash warning is one of the main systems of public safety applications, which is used to prevent the occurrence of possible accidents. Due to an accident, a disabled vehicle sends warning messages to the other vehicles traveling on the same route by using V2V and V2I communications to share the incident location, direction, and route information [32].

#### 7.2.2. Sign Extension

The aim of this application is to notify the careless drivers for signs, which are assembled on the roadside in order to prevent drivers from an accident. Applications of sign extension works on 1 Hz on I2V communication and use the safety messages with a communication range, which varies from 100 to 500 m [32]. The sign extension application can be further classified as follows.

- In-Vehicle Signage: In this application, RSU is fixed on the specific location i.e., hospital area, school zone, and university area to broadcast safety messages to vehicles approaching those locations.
- Turning Speed Warning: In this application, RSU is fixed on the road before turning to broadcast the safety messages to vehicles approaching toward turning. The speed of vehicles needs to be discussed with turning in order to safely pass through the turning.
- Low-Bridge Warning: This application is used to alert the driver for the height of the bridge by broadcasting warning messages to the vehicle through road infrastructure, which is assembled in the vicinity of the bridge. Additionally, an OBU is equipped in the vehicle, which can determine whether or not there is a space to pass through the bridge.
- In-Vehicle Amber Alert: This application relies on I2V communication and sends (Americas broadcast emergency response) messages to vehicles. This message is only broadcast when the law enforcement authorities confirm that the vehicle is involved in suspected crime, and circulate amber alerts to the vehicle in that area excluding a suspected vehicle.
- Low-Parking Warning: This application notifies the driver for the minimum car parking height in which the vehicle is trying to enter. This system relies on broadcasting warning messages to the vehicle from an RSU, which is placed near the parking lot, and then OBU can analyze whether or not to enter in the parking area.

#### 7.2.3. Information from Other Vehicles

These applications depend on the V2V and V2I communications with the operating frequency of 2 to 50 Hz along with the message requiring a communication range of 50 to 400 m. This application is used to improve the traffic flow, ensure the safety of the passengers by accurately identifying the traffic conditions, and assist drivers in taking an alternate route in advance. This application is further classified, as follows.

- Cooperative Forward Collision Warning: This application guides vehicles to take the alternate route in order to avoid travel to the accident location. This application uses V2V communication with a multi-hop technique to send warning messages to the driver regarding the incident situation. Warning messages include position, direction, route, speed, etc. so that each vehicle on the road can analyze the severity of an incident and then forward it to other vehicles.
- Road Condition Warning: This application relies on V2V communication. In this application, vehicles obtain sufficient information about the road conditions from the electronic sensors equipped in the vehicle. After obtaining the information, the TA process this information to identify the road conditions and send the warning messages to the driver and the other vehicles on the road.
- Pre-Crash Sensing: This application aims to predict the situation in which an accident is about to happen. The information can be collected from vehicle sensors, and additional information may be required from other vehicles via V2V communication in order to predict a situation. This application is most helpful for heavy traffic and aims to enhance the safety of passengers and drivers.
- V2V Road Feature Notification: This application is used to collect information for the road infrastructure by using V2V communication, and then broadcasting this information to the other vehicles on the road. By sending messages, this application assists and notifies drivers of the road conditions such as potholes, debris, etc. in advance, and then the alerted vehicle sends this notification to other vehicles on the road. This application is most applicable in rural traffic where road surfaces are not smooth.
- Emergency Electronic Brake light: In case of smog or foggy weather, this application warns the vehicles traveling on the road for sudden hard breaking by using V2V communication that broadcast safety messages to the other vehicles, and notify them for hard breaking in advance.

#### 7.2.4. Vehicle Diagnosis 

This application sends a reminder to the drivers in the form of messages for vehicle safety defects. Once the vehicle received this notification, they start to process for maintenance. This application utilizes I2V communication with the safety messages requiring a communication range of 400 m. This application is further classified below.

- Safety Recall Notice: In this application, a message is sent to the drivers for reminding a safety recall issuance.
- Just-in-time Repair Notification: In this application, there is a fault existing in the vehicles. First, the OBU broadcast a message to the RSU by using V2I communication. Then, the vehicle will receive an instruction message from TA to handle these issues by I2V communication. This application is useful to alleviate the traffic incident and congestion in an urban traffic environment.

#### 7.2.5. Intersection Collision Avoidance 

This system relies on V2I communication in which RSU collect and process traffic-related information from the vehicle approaching close to the intersection and then forward this information to the TA. Then, TA determines, if the probability of occurrence of an incident at intersection is high, then TA sends a warning message to the vehicles in the intersection area to take precautionary measures about the possibility of an accident. This system significantly reduced the traffic incident by analyzing and obtaining an accurate estimation of the possibility of an incident occurring. In particular, this application provides a sophisticated tool in the ITS for reducing traffic incident and congestion. 

This application uses I2V communication with the frequency of 10 Hz and the safety messages with a communication range of 200 to 300 m. This application is further classified below.

- Warning for Violating the Stop Sign: In this application, a warning message is sent to the vehicles to warn the driver about the distance between the vehicle and the stop sign and also the speed necessary to avoid hard breaking. This application reduces the violation of stop signs, which can prevent the vehicle from a fatal accident or hazardous conditions.
- Violating Traffic Signal Warning: This application is used to send warning messages to the vehicles and warn them about a hazardous condition, i.e., fatal accident, severe incident, road bottlenecks, etc. that may happen if the vehicle did not stop in front of the sign [167].
- Intersection Collision Warning: This application gathers the information of the road intersection through the electronics sensors and then analyzes this information. The system will send a warning message to the vehicles in case of probability of an incident occurring is high. In this application, the data collected by the sensors include speed, position, acceleration, traffic flow information, etc.
- Pedestrian Crossing Information: This application is used to notify the driver if there is a pedestrian crossing the road. In this application, the information is collected for the pedestrians through sensors installed in the sidewalk, which can significantly reduce accidents by sending alert messages to the vehicles instantaneously in case a potential collision between pedestrians and vehicles is identified.

## 8. Open Research Challenges

It has been observed from the literature reviewed that many challenges still exist in the VANET security and requires research and investigation in order to design sophisticated algorithms, which can tackle all security challenges. The open research challenges are discussed below.

### 8.1. Challenge on Security

Since the communication takes place in an open access environment, which make VANETs more vulnerable to attacks that cause multiple threats to the security services. In particular, a Sybil attack is one of the most dangerous attacks in VANETs. This attack is the most influential attack that is addressed by the researchers. Maintaining the balance between privacy and non-repudiation is still required in order to detect Sybil attacks. In the past, many studies have been done on Sybil attacks. On the other hand, many other attacks such as jamming attacks and malware attacks in VANETs motivate further research. Many security challenges still exist, which need superior algorithms in order to solve these challenges. A robust authentication technique along with the message exchange approach is used to protect V2I and V2V communications from inside and outside attackers. The main problem occurs when the utilization of CA and TA and the schemes that rely on the private keys for V2X, i.e., V2V and V2I communications, can protect the vehicular network. However, it can increase the computational and transmission overheads and introduce the scalability challenges.

Kang et al. [168] presented an access control technique for message broadcasting in VANETs, which aim to protect the message privacy. However, this scheme did not consider communication overhead and the delay occurred in the verification process. Wasef and Shen [115] introduced an EMAP technique for VANETs. This scheme alleviated the large computational process of CRL by an effective revocation process. This scheme significantly reduced the authentication delay. However, this scheme did not consider the communication overhead and cost occurred during the authentication process. In Reference [123], Azees et al. introduced an EAAP scheme to avoid a malicious vehicle from entering in VANETs. However, in this scheme, the signature verification can produce a delay in the verification process. Jiang et al. [169] introduced an efficient anonymous batch verification technique. This scheme somehow improves the efficiency of VANETs by utilizing the hash message authentication code (HMAC). However, it increases the packet loss. 

More specifically, VANET security remains a challenging issue, in which the authentication scheme plays an important role for tackling all kinds of threats and attacks. In this context, we still need an efficient authentication scheme, i.e., message authentication to protect the V2V and V2I communications.

### 8.2. Challenge on Routing

Routing protocols play an important role in VANETs. Since the high mobility vehicles in VANETs influence the changing network topology. Such an influence challenges the routing protocol in VANETs. In the past, several research works conducted the research on routing protocols and identified some limitations such as large computational processing, routing un-scalability, routing services, and cost [170,171]. This problem motivates the research community to introduce robust scalable routing protocols that can alleviate the above-mentioned issues. In Reference [172], Li et al. proposed a new routing protocol for VANETs to adaptively select the intersections, in which the data packet delivery and the routes that are selected must satisfy the quality of service (QoS) constraints. Khan et al. [173] presented a novel traffic aware segment based routing (TASR) protocol by considering the vehicle density and delivery ratio in order to transmit packets and obtain a better routing protocol for urban mobility. Ding et al. [174] presented a traffic light aware routing protocols named as a street centric protocol to obtain the enhanced delivery ratio and minimize end-to-end delay for an urban traffic environment in VANETs. 

In particular, the reliability of successfully broadcasting the traffic information among vehicles can enhance driving experiences, allow drivers to analyze the current traffic conditions, and take safety measures, which can significantly improve the traffic and road safeties. Such safety systems are one of the main challenging issues that exist in VANETs.

### 8.3. Challenge on a Reliable Link

The next challenging issue in VANETs is the lack of reliable links between V2V and V2I to collect traffic-related data. In the past, several research studies have been done to detect malicious users, but suffers with the lack of a reliable link between entities due to high mobility on the road. Therefore, the high mobility remains a challenging issue in VANETs. 

Reliable links between entities improves the detection of malicious users. Developing an intrusion detection mechanism could enhance the detection rate by incorporating different wireless technologies. However, at this stage, intrusion detection has not been well studied and implemented in this domain.

### 8.4. Challenge on Group Signature

In VANETs, the group signature is a robust authentication technique, which is used to provide secured communication in a vehicular network. However, the key distribution group of a high-speed moving vehicle still remains a challenging issue. In Reference [150], Malina et al. introduced a group signature with short-term linkability and categorized the batch verification. This scheme produced an efficient signing mechanism and provided a secure mechanism to register and revoke users. However, this mechanism required a longer time to solve the batch verification when a large number of malicious messages was presented in the batch. In Reference [175], Sampigethaya et al. introduced a scheme they call AMOEBA to provide location privacy by using a group of vehicles. This scheme enables all members of the group to send a message anonymously to the group manager. This method protects user privacy by ignoring the group manager. However, AMOEBA has a main concern in which the information such as location, id, etc. can be disclosed by the group leader. Zhang et al. [151] introduced an LBS protocol, in which the proposed method is able to provide different security services to the vehicle user and service provider for each transaction. This method ensures vehicle privacy by providing service to the vehicle anonymously. However, in case of heavy vehicle density, each LBS event may introduce large computational overhead.

From the above schemes, we analyze that the researcher from the wireless communication technology background should focus on the VANET security services and scalability of key distribution schemes in group-based signature authentication.

## 9. Conclusions

The VANET is a vital and promising research area in ITS due to high mobility and dynamic network topology. It aims to ensure the safety of human lives on the street by broadcasting safety messages among the vehicles and provide comfort services to the passengers. Since the safety messages are broadcasted in an open access environment, VANETs are vulnerable to attacks. Therefore, sophisticated and robust security algorithms must be designed to tackle the dangerous security and privacy attacks. 

This survey provides a comprehensive review and covers most of the issues of VANETs. First, we have discussed the basic model and function of the VANET. Then, the security services as well as threats and attacks on these services are explained, followed by the recent state-of-the-art schemes on each security service. Second, we have comprehensively covered the authentication schemes in detail, which are able to protect the vehicular network from malicious nodes and fake messages. Third, we have discussed the various simulation tools and the performance of the authentication schemes in terms of simulation tools, which was followed by the VANET applications. Lastly, we identified some open research challenges and future research directions in VANETs. In short, this survey is well developed to cover most aspects in security issues and privacy-preserving methods, while filling the gaps of existing surveys and incorporating the latest trends in VANETs.

As the importance and popularity of V2X, C-V2X and LTE-V communications in the ITS increases, the traffic information such as vehicle ID, location, and the weather conditions can be shared between all vehicles. The drivers and passengers not only look for the reliable and trustworthiness of the large amount of exchange information required for privacy protection, but also look for the robust authentication algorithms that are able to protect VANETs from different kinds of threats and attacks. More specifically, there is a great need for designing VANET algorithms in order to tackle all these challenges. Such algorithms can provide trustworthy communication on V2V and V2I, and also protect vehicle ID and location privacy. The future research directions for the VANET system should focus on security and privacy issues such as privacy preservation, trust management, location privacy, and so on. Such research directions require more time to study and conduct research in order to design and develop robust algorithms, which can tackle different kinds of security threats and challenges. In addition to these directions, the security systems must be enhanced by efficient authentication schemes for providing a secure communication. Additionally, an efficient intrusion detection system could be incorporated in vehicular networks to detect the malicious nodes and handle all types of security attacks.
